# Duration of intermittent hypoxia impacts metabolic outcomes and severity of murine NAFLD

**DOI:** 10.3389/frsle.2023.1215944

**Published:** 2023-08-25

**Authors:** Laura A. Barnes, Yinuo Xu, Ana Sanchez-Azofra, Esteban A. Moya, Michelle P. Zhang, Laura E. Crotty Alexander, Atul Malhotra, Omar Mesarwi

**Affiliations:** 1 Division of Pulmonary, Critical Care, and Sleep Medicine and Physiology, School of Medicine, University of California, San Diego, San Diego, CA, United States,; 2 School of Biological Sciences, University of California, San Diego, San Diego, CA, United States,; 3 Division of Pulmonary and Sleep Medicine, Hospital Universitario de la Princesa, Universidad Autónoma de Madrid, Madrid, Spain,; 4 Section of Pulmonary and Critical Care, VA San Diego, La Jolla, CA, United States

**Keywords:** liver fibrosis, diet induced obesity, metabolism in hypoxia, metabolism in obstructive sleep apnea, NAFLD

## Abstract

**Rationale:**

Obstructive sleep apnea (OSA) is associated with metabolic dysfunction, including progression of nonalcoholic fatty liver disease (NAFLD). Chronic intermittent hypoxia (IH) as a model of OSA worsens hepatic steatosis and fibrosis in rodents with diet induced obesity. However, IH also causes weight loss, thus complicating attempts to co-model OSA and NAFLD. We sought to determine the effect of various durations of IH exposure on metabolic and liver-related outcomes in a murine NAFLD model. We hypothesized that longer IH duration would worsen the NAFLD phenotype.

**Methods:**

Male C57BL/6J mice (*n* = 32) were fed a high *trans*-fat diet for 24 weeks, to induce NAFLD with severe steatohepatitis. Mice were exposed to an IH profile modeling severe OSA, for variable durations (0, 6, 12, or 18 weeks). Intraperitoneal glucose tolerance test was measured at baseline and at six-week intervals. Liver triglycerides, collagen and other markers of NAFLD were measured at sacrifice.

**Results:**

Mice exposed to IH for 12 weeks gained less weight (*p* = 0.023), and had lower liver weight (*p* = 0.008) relative to room air controls. These effects were not observed in the other IH groups. IH of longer duration transiently worsened glucose tolerance, but this effect was not seen in the groups exposed to shorter durations of IH. IH exposure for 12 or 18 weeks exacerbated liver fibrosis, with the largest increase in hepatic collagen observed in mice exposed to IH for 12 weeks.

**Discussion:**

Duration of IH significantly impacts clinically relevant outcomes in a NAFLD model, including body weight, fasting glucose, glucose tolerance, and liver fibrosis.

## Introduction

Obstructive sleep apnea (OSA) is a highly prevalent disorder characterized by repetitive airway closure during sleep (Dempsey et al., 2010). OSA has been linked to a variety of metabolic disorders including dysglycemia and incident type 2 diabetes mellitus, atherosclerosis, and nonalcoholic fatty liver disease (NAFLD) (Mesarwi et al., 2019). NAFLD is a hepatic manifestation of the metabolic syndrome, and is defined by the presence of liver steatosis without other causes of liver injury, such as viral hepatitis, drug induced liver injury, or significant alcohol consumption (Byrne and Targher, 2015). Worldwide, NAFLD affects up to 30% of the population (Bhala et al., 2013), and a recent analysis of the national health and nutrition examination survey (NHANES) data showed that the prevalence of NAFLD in the general U.S. population is nearly 40% (Ciardullo and Perseghin, 2021). In some patients with NAFLD, there will be progression of the phenotype of liver steatosis to one of overt inflammatory injury and liver fibrosis. In those who develop nonalcoholic steatohepatitis (NASH) due to NAFLD, there is a high risk for progression to liver failure, need for organ transplantation, and/or death (Loomba and Sanyal, 2013). Thus, NAFLD is a major cause of morbidity and mortality in the U.S. and worldwide.

Some progress has been made toward a mechanistic understanding of the complex interaction between OSA and NAFLD. NAFLD severity appears to be linked to the degree of hypoxemia in OSA, with mean and nadir nocturnal oxyhemoglobin saturations correlating with increased risk of NAFLD progression, as well as time spent with oxyhemoglobin saturation <90% (Mishra et al., 2008; Polotsky et al., 2009; Türkay et al., 2012; Cakmak et al., 2015; Qi et al., 2016; Trzepizur et al., 2016). These findings have been demonstrated in general OSA patient populations (Türkay et al., 2012; Cakmak et al., 2015; Trzepizur et al., 2016), in the severely obese (Mishra et al., 2008; Polotsky et al., 2009), and even in those of normal weight (Qi et al., 2016). Rodent models of OSA have been developed using chronic intermittent hypoxia (IH) to model the hypoxemia of OSA (Barnes et al., 2022), and various models of NAFLD in rodents have been used, including high-fat diets. Mice exposed to IH develop liver fibrosis and hepatic inflammation and oxidative stress (Savransky et al., 2007a; Jun et al., 2008; Kang et al., 2017; Mesarwi et al., 2021), and exposure to IH in obese mice results in elevated hepatic triglycerides and histologic evidence of NAFLD (Drager et al., 2011). Prior studies have shown that a high *trans*-fat diet induces hepatic steatosis (Clapper et al., 2013; Kristiansen et al., 2016). We have previously shown that, in a high trans-fat diet model of murine NAFLD, both progression of liver fibrosis and expression of lipogenic genes are mediated by hepatocyte hypoxia inducible factor-1 (HIF-1) (Mesarwi et al., 2016). We have also shown that IH modeling severe OSA enhances liver fibrosis in murine NAFLD, likely by exacerbating inflammatory injury, but that IH may act via HIF-1-independent paths to account for these changes (Mesarwi et al., 2021). However, to our knowledge it has not previously been investigated whether different durations of IH in a NAFLD model can variably worsen liver fibrosis or other histological findings in NAFLD. In this study, we aimed to characterize the effects of variable durations of IH in an established NAFLD model, in order to determine whether IH of longer duration results in more severe steatohepatitis and fibrosis in an established NAFLD model, and to define ideal co-modeling of these complex diseases. We hypothesized that IH of longer duration would result in a more severe NAFLD phenotype (worsened liver fibrosis and hepatic steatosis), relative to IH of shorter duration.

## Materials and methods

### Animals

Male C57BL/6J mice, age 8 weeks, *n* = 8/group, were fed a high *trans*-fat diet (HTFD; 40% fat, 18% of which was *trans*-fat; 22% fructose; and 2% cholesterol; Research Diets, Inc.) to induce NAFLD and NASH. Animals were exposed to light from 7 a.m. until 7 p.m. daily, housed at four mice per cage, and had food intake and weight recorded twice weekly. At the time of death, serum, liver, and epididymal fat were collected. Animal studies were approved by the Institutional Animal Care and Use Committee of the University of California, San Diego.

### Experimental procedure and hypoxia exposure

**FIGURE 1:**
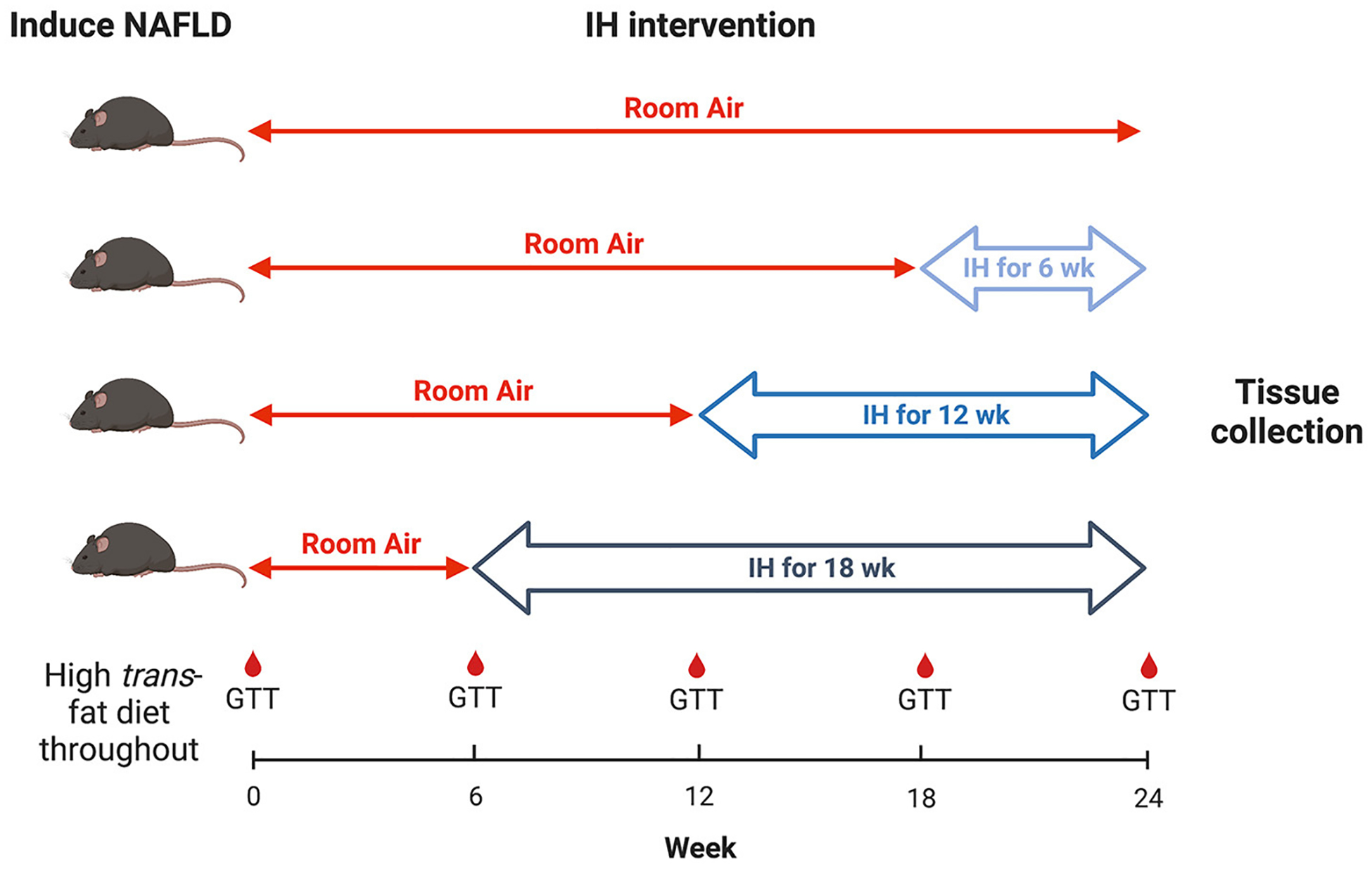
Experimental design. Timeline of hypoxia initiation and GTT intervals.

All mice were continued on the HTFD for 24 weeks. At week 6 from the start of the HTFD, one group of mice were started on IH. At week 12, a second group were started on IH. At week 18, a third group were started on IH, and a fourth group was kept in room air (RA) as a control. Thus, the groups were exposed to IH for 18, 12, 6, and 0 weeks, respectively, in order to understand the effect of different durations of IH on NAFLD progression (Figure 1). For IH, a programmable device (Hycob 4 channel Hypoxia System, Technolutions, New Plymouth, NZ) allowed adjustment of the gas content in custom cages to fluctuate between a fraction of inspired oxygen (FiO_2_) of 0.21 and an FiO_2_ of 0.06 once per min for 12 h per day during the light phase. RA exposure worked similarly, so as to control for the continuous flow of gas into the cages, but air (FiO_2_ of 0.21) was supplied continuously.

### Glucose tolerance tests

At the start of the experiment and every 6 weeks thereafter, intraperitoneal glucose tolerance tests (GTTs) were performed. Mice were fasted for 5 h (Ayala et al., 2010), beginning at 7:30 a.m. A basal glucose level was obtained by using the tail scratch technique at time 0 with a handheld glucometer (ACCU-CHEK Guide, Roche), and the mice were injected intraperitoneally with 1 g/kg glucose. Blood glucose was checked at 15, 30, 45, 60, 90, and 120 min after glucose injection. GTT results were analyzed by subtracting fasting glucose levels and calculating the area under the curve (AUC) for each mouse. HOMA-IR, a metric of insulin resistance, was calculated with the following formula: HOMA-IR = fasting glucose (mg/dL) x fasting insulin (μU/mL)/405.

### Sacrifice

Mice were deeply anesthetized with isoflurane; tail pinch was used to ensure appropriate depth of anesthesia. The abdominal cavity was opened and an incision was made into the aorta, for exsanguination and to collect blood for serum assays. The liver was excised carefully, weighed, and part was flash frozen in liquid nitrogen and another section was kept in 10% formalin for histologic evaluation. Epididymal fat was collected similarly.

### Liver and serum assays

Fresh liver tissue samples were collected in 10% buffered formalin and then dehydrated with 70% ethyl alcohol after 24 h and embedded in paraffin. Liver tissue was sectioned into 5-μm slices for staining. Hematoxylin and eosin (H&E) stains and Sirius red stains were used to characterize NASH and liver fibrosis. Collagen was quantified by using a hydroxyproline assay (QuickZyme Biosciences). Collagen content was determined from this assay by assuming a hydroxyproline content of 13.5% (Neuman and Logan, 1950), and samples were normalized for liver weight. Whole-liver tissue was homogenized, the triglyceride content was determined (Cayman Chemical Co.), and malondialdehyde (MDA) was assessed (BioAssay Systems).

### Statistical analysis

Between-group comparisons for data with single-point measurements were made by one-way ANOVA. *Post-hoc* comparisons were made with Tukey’s multiple comparisons test. For all statistical analyses, a *p* value of <0.050 (or adjusted *p* value, in the *post-hoc* comparisons) was the threshold used for statistical significance. Data are reported as the mean ± SEM unless otherwise noted. Prism 9 software (GraphPad, San Diego, CA) was used for all analyses.

## Results

### Body weight and distribution

**FIGURE 2:**
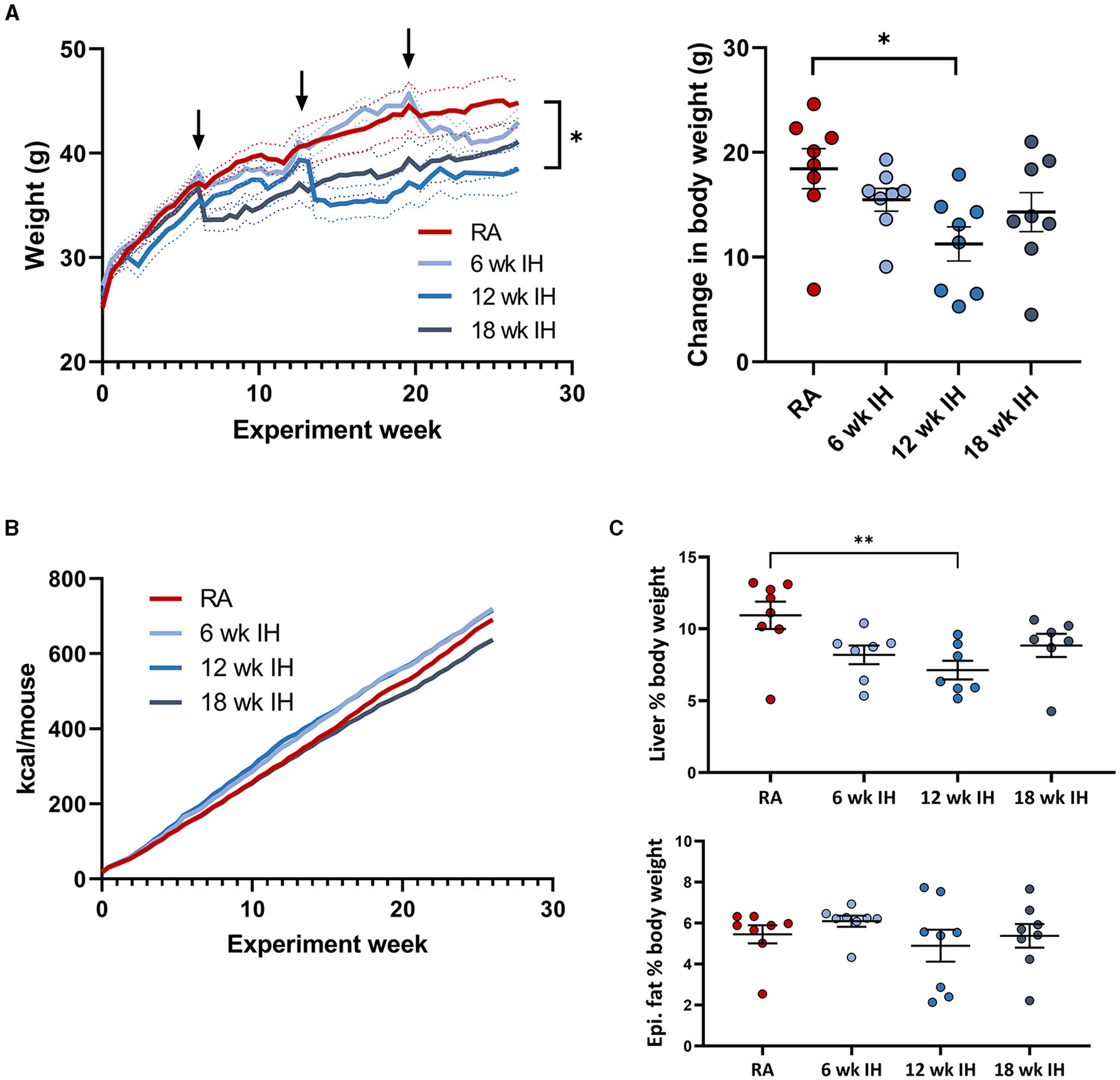
Weight, food intake, and body composition. **(A)** (left) Weight over the experiment duration. Mice gained weight on the high fat diet, but lost weight in response to IH (arrows indicate IH exposure). (Right) Change in body weight over experiment duration. **(B)** Cumulative food intake in each group. **(C)** Liver and epididymal fat weight at the time of sacrifice. *p < 0.05; **p < 0.01 relative to RA group.

As expected, mice gained weight over the experiment duration, as they were all on a high fat diet (Figure 2A; arrows indicate when successive groups began exposure to IH). Mice in each IH group lost weight immediately after IH exposure began, but generally returned to the same pace of weight gain thereafter. We also observed a more prolonged period of weight loss in response to IH as IH exposure was delayed. Mice exposed to IH for the final 6 weeks of the experiment reached a nadir weight in response to IH at 28.1 ± 2.7 d, vs. 20.1 ± 3.6 d (*p* = 0.019 relative to the 6 week IH group), and 8.8 ± 2.0 d (*p* < 0.001 relative to the 6 week IH group). There was a significant effect of hypoxic exposure on total weight gain (*p* = 0.037 by one-way ANOVA), though only the 12 week IH group was different from RA controls in *post-hoc* analysis (11.3 ± 1.6 g vs. 18.5 ± 1.9 g, respectively, *p* = 0.023). Cumulative food intake over the experiment duration is shown in Figure 2B. Food intake was determined per cage of four mice, precluding statistical analysis. We did not observe significant plateaus of the food intake curves in any group in response to IH.

Liver weight was lower in mice exposed to IH for 12 weeks relative to RA, without other between-group differences (Figure 2C, RA: 4.88 ± 0.57 g; 6 week IH: 3.47 ± 0.34 g [*p* = 0.137]; 12 week IH: 2.65 ± 0.37 g [*p* = 0.008]; 18 week IH: 3.62 ± 0.44 g [*p* = 0.209]). Liver as a percentage of total body weight at the time of sacrifice was also reduced in 12 week IH relative to RA (RA: 10.9 ± 0.9%, 12 week IH: 7.1 ± 0.7%, *p* = 0.010), without significant effects in the other IH groups relative to RA. Epididymal fat weight was not different between groups.

### Glucose, insulin, insulin resistance

**FIGURE 3:**
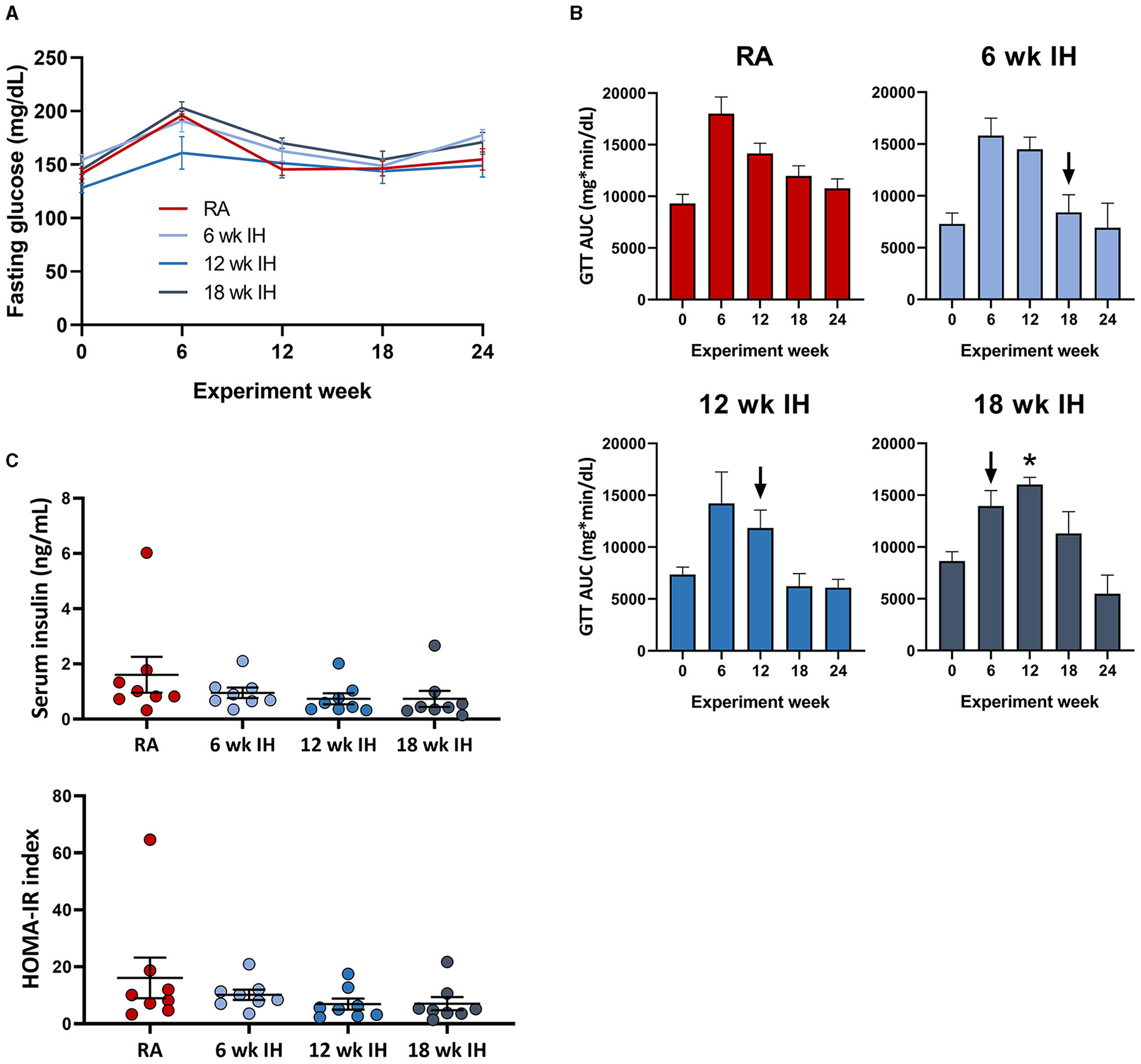
Glucose metabolism and insulin levels. **(A)** Fasting glucose over time. **(B)** Glucose tolerance tests over time. Arrows indicate initiation of hypoxic exposure in each group. **(C)** Insulin and HOMA-IR at the time of sacrifice. *p < 0.05; for comparison of GTT AUC pre- and post-IH initiation.

Fasting glucose was assessed as the time point 0 glucose level during serial GTTs. IH did not significantly impact fasting glucose levels over time, irrespective of when IH was initiated (Figure 3A); there was only one significant between-group difference at one time point (12 week IH vs. 18 week IH at week 6 GTT, *p* = 0.026); all other comparisons at all other time points were not significant. The dynamic response to glucose load was assessed during GTT (Figure 3B). In general there was a trend of initial increase in the GTT AUC as the mice were started on the HTFD, but the AUC then trended down over time. We examined the response to IH by comparing in each IH group the GTT AUC just prior to, and 6 weeks after, IH initiation (ΔGTT). This value was significantly different in the mice exposed to IH for 18 weeks relative to mice in IH for 12 weeks (*p* = 0.013) and mice in IH for 6 weeks (*p* = 0.044).Insulin levels at the time of sacrifice and HOMA-IR were similar between groups (Figure 3C).

### Liver characteristics

**FIGURE 4:**
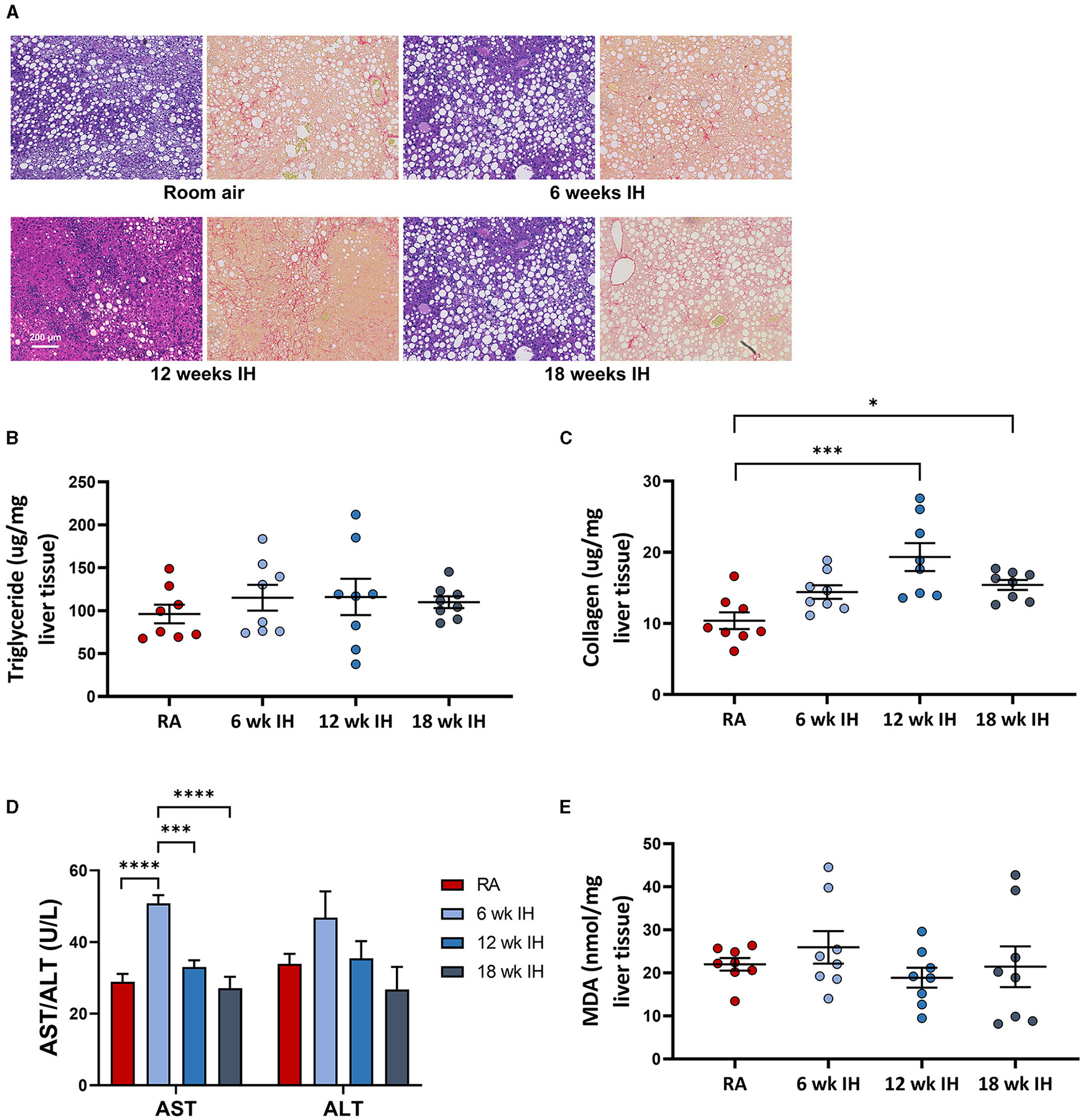
NAFLD characteristics. **(A)** Representative liver histology in each group (left, Mason’s trichrome stain, and right, Sirius red stain for collagen). **(B)** Liver triglycerides at sacrifice. **(C)** Hepatic levels of collagen as measured by hydroxyproline assay. **(D)** Serum aminotransferases at sacrifice. *p < 0.05; ***p < 0.001; ****p < 0.0001. **(E)** Liver malondialdehyde at the time of sacrifice.

Representative images from liver histology are shown in Figure 4A. Liver triglycerides at sacrifice were similar between groups (Figure 4B). Liver collagen, as measured by hydroxyproline assay, was different as a function of hypoxia (*p* < 0.001 per one-way ANOVA, Figure 4C). In RA, collagen concentration was 10.4 ± 1.2 μg/mg liver tissue; in 6 week IH, it was 14.4 ± 1.0 μg/mg liver tissue (*p* = 0.145); in 12 week IH, it was 19.3 ± 2.0 μg/mg liver tissue (*p* < 0.001); and in 18 week IH, it was 15.4 ± 0.7 μg/mg liver tissue (*p* = 0.048). Serum AST at sacrifice was higher in the 6 week IH group relative to all other groups, and ALT was similar between groups (Figure 4D). Liver MDA (Figure 4E) was similar in all four groups: 21.9 ± 1.5 μM/mg tissue in the RA group; 25.9 ± 3.7 in the 6 week IH group (*p* = 0.832 relative to RA); 18.9 ± 2.3 in the 12 week IH group (*p* = 0.912); 21.4 ± 4.7 in the 18 week IH group (*p* = 0.999).

## Discussion

This experiment was designed to determine the effect of different durations of IH on metabolic and liver-related outcomes in a mouse model of NAFLD, with the aim to optimize co-modeling OSA and NAFLD. There have been many recent investigations into the mechanistic underpinnings linking these two disease states (Aron-Wisnewsky and Pepin, 2015; Kang et al., 2017; Chen et al., 2020; Mesarwi et al., 2021; Zhang et al., 2021; Hazlehurst et al., 2022; Tan et al., 2022). Prior studies, including our own (Mesarwi et al., 2021), have typically employed shorter, 4–6 week durations of IH, though some studies have examined the effects of IH of longer duration (e.g., 6 months) (Savransky et al., 2007a). In this experiment, we found differences in the severity of liver fibrosis as a function of CIH duration, with a peak in liver collagen content at 12 weeks of IH. In addition, we saw differences in glucose metabolism and body composition depending on IH exposure duration, without clear effects on liver triglycerides.

There is complexity in co-modeling hypoxia and NAFLD, since hypoxia (sustained or intermittent) generally leads to weight loss, particularly in obese animals (Barnes et al., 2022). Savaransky et al. found that 6 months of IH exposure completely prevented weight gain in a diet-induced obesity NAFLD model (Savransky et al., 2007a). In our study, we did see acute weight loss after each group was placed into IH, and long-term, mice exposed to IH for 12 weeks gained less weight than those in RA, though there were no differences in the other groups, and in general weight gain resumed after acute loss. We note two interesting findings about weight changes across different durations of IH: first, there was a significant difference in the amount of time needed to reach nadir weight after IH initiation, with more delayed IH resulting in a more prolonged “catch up” period. Second, changes in body weight in response to IH did not appear to be explained by differences in food intake alone, as we saw no plateau of the cumulative caloric intake curve concurrent with the introduction of IH in any group. This finding strongly suggests that the global metabolic rate increased with IH, irrespective of when it was introduced. Whether this result was due to increased activity or other metabolic changes is unclear, though anecdotally we have observed *reduced* activity levels in mice acutely placed into IH. Moreover, epidydimal fat mass, which provides a rough estimate of overall animal adiposity, was not different between groups. This result suggests that any differences in other outcomes (e.g., hepatic collagen) were likely not driven by overall changes in obesity. Additionally, IH is known to induce changes to plasma free fatty acid levels, lipoprotein lipase activity, and serum triglycerides (Drager et al., 2012; Jun et al., 2012; Yao et al., 2013), and any of these effects may complicate the findings we report and are worthy of future investigation.

Liver fibrosis is the most clinically important outcome in the study of NAFLD, as fibrosis in humans is uniquely linked to need for transplantation and early liver-related death (Treeprasertsuk et al., 2013; Angulo et al., 2015; Tada et al., 2017; Unalp-Arida and Ruhl, 2017). There are many possible mechanisms for IH to induce liver fibrosis, including via oxidative stress, hypoxia inducible factor mediated inflammation, induction of the TLR4/MAPK/NFkB pathways (Kang et al., 2017), and while this study is not designed to be mechanistic, our findings may inform future studies. We observed IH duration-dependent differences in the degree of liver fibrosis (as measured by liver collagen), but not in other NAFLD-related outcomes, such as hepatic triglyceride content. Though AST levels were elevated in mice in IH for 6 weeks, the context of this is unclear as the values were generally less than what we have observed previously, and increased levels were not observed in other groups, or mirrored by ALT levels. In our previous work, we observed that 6 weeks of IH (similar to the 6-wk IH group in the present experiment), increased liver fibrosis in mice on the same high *trans*-fat diet (Mesarwi et al., 2021). We observed this trend in the current work, but liver fibrosis was worse still in mice exposed to longer durations of IH, with a peak in the 12 week group. To us, this informs study design to examining IH effects on NAFLD, and suggests that future experiments use longer durations of IH if liver fibrosis is the outcome of interest.

The observation of the highest magnitude of liver collagen in mice which gained the least weight overall is curious. On one hand, this finding seems counterintuitive, as NAFLD development and progression is clearly related to obesity. However, both epididymal fat and HOMA-IR were similar in all groups, suggesting that the relationship between obesity and liver cirrhosis in this model relies on more factors than weight gain alone. In fact, in humans with NAFLD, several studies have noted that visceral adiposity is associated with poor NAFLD outcomes, and in some cases, this association may be stronger than BMI (Yu et al., 2015; Lee et al., 2017; Xu et al., 2018). The same may be true in OSA; studies have noted that visceral fat is more strongly associated with OSA severity than subcutaneous fat, or total body fat, and in men the association seems particularly strong (Kritikou et al., 2013; Harada et al., 2014; Ma et al., 2022). Our study highlights the complexity of the interactions between weight, OSA, and NAFLD, and more work is clearly indicated.

In our study, we did not see a difference in hepatic triglyceride content at any duration of IH, which is in concordance with our prior work examining 6 weeks of IH. Drager et al. found that in mice on a different high fat diet, IH induced excess hepatic triglycerides in just 4 weeks of IH (Drager et al., 2011). This same effect was not observed with very long IH duration (i.e., 6 months) (Savransky et al., 2007a). We suggest that parameters such as the precise fat content and composition of the diet, and duration of the high fat diet, may play a more important role in the determination of IH effect, than IH itself. Similarly, previous studies have shown fairly clearly that IH induces dysglycemia in lean and obese mice (Drager et al., 2011; Zhen et al., 2021), an effect we did not observe, whether due to a ceiling effect of the HTFD, or other cause. However, we note that our previous work on the effects of IH in a similar NAFLD model did not demonstrate IH effects on glycemia (GTT AUC, fasting glucose, fasting insulin, or HOMA-IR). Again, the deleterious effect of the diet may far outweigh IH effects. Thus, any future studies examining glucose metabolism as a primary outcome may best be designed around a less severe NAFLD model.

Our study has some important limitations. First, IH does not perfectly recapitulate all the physiological effects of OSA, and hypercapnia, intrathoracic pressure swings, and sleep fragmentation, all observed in some studies to have metabolic effects independent of IH, are not modeled here. Nonetheless, this does not preclude effects from IH alone, and IH is clearly an important manifestation of OSA. Second, though the mice used in this experiment were genetically similar and of similar weight and age at baseline, there were minor (though not statistically significant) differences in baseline glucose levels in the mice exposed to IH for 12 weeks, for unclear reasons. However, we consider it somewhat unlikely that a lower baseline glucose level might have contributed to enhanced liver fibrosis. Third, while this study focuses on the duration of exposure to intermittent hypoxia, the duration of the diet used to induce NAFLD is also important, and by experimental design, the time on this diet varied between groups prior to IH. Secondary studies assessing the effects of this diet in varying duration may be instructive. Moreover, the decision to introduce IH after, rather than before or concurrent with the HTFD, was based on the desire to understand the interaction between superimposed IH on a well-established NAFLD model. In so doing, we intentionally did not use lean or chow diet groups in this study. Although numerous studies have documented various metabolic effects of IH in lean mice, even 12 weeks of IH in a similar regimen as used in our study did not induce demonstrable liver steatosis or inflammatory injury (Savransky et al., 2007b), in contrast to our findings in an obese model. Fourth, we note that there may be an age effect in this experiment that is unexplored, as again mice were put into IH at different time points. Fifth, we did no examine the effect of IH or HFD on the pancreas, though IH has been shown to alter pancreatic function and changes in glycemia may influence NAFLD progression (Yokoe et al., 2008). Finally, we note that the diet used in this experiment is unfortunately no longer available in the U.S. due to federal limitations in the use of *trans*-fats in food products. We applaud this move, designed to improve public health, but note that a very similar diet, using palm oil as a fat source, has been made available and has been shown to have similar hepatic effects.

In conclusion, IH at variable duration has varying metabolic effects in a mouse model of NAFLD. When designing experiments aimed at co-modeling OSA and NAFLD, the ideal experimental design may depend on the outcome of interest. Studies primarily examining liver fibrosis as an outcome, which we suspect will be the majority, would be best designed by aiming for 12 weeks of IH concurrent with the diet used in this experiment, or similar depending on availability. Experiments with glucose metabolism as a primary outcome may be optimally designed with different IH duration.

## Data Availability

The raw data supporting the conclusions of this article will be made available by the authors, without undue reservation.
